# Clinical Evaluation of an AI-Assisted Decision Support System for General Anesthesia Management Based on Data From 6 Centers: Comparative Study

**DOI:** 10.2196/90023

**Published:** 2026-07-20

**Authors:** Dongxu Chen, Qingsheng Xue, Geng Wang, Shanshan Mu, Zhen Zeng, Bin Xu, Shiyue Li, Yu Chen, Weidong Gu, Jing Shi

**Affiliations:** 1 Department of Anesthesiology, Sichuan Academy of Medical Sciences & Sichuan Provincial People's Hospital, University of Electronic Science and Technology of China Chengdu, Sichuan China; 2 Ruijin Hospital Shanghai, Shanghai China; 3 Beijing Jishuitan Hospital of Capital Medical University Beijing China; 4 Beijing Chaoyang Hospital of Capital Medical University Beijing China; 5 Shanghai Sixth People Hospital affiliated to Shanghai Jiao Tong University School of Medicine Shanghai China; 6 Shanghai Ruiyi Wei Medical Technology Co., Ltd. Shanghai China; 7 West China Hospital of Stomatology, Sichuan University Chengdu China; 8 West China Fourth Hospital, Sichuan University Chengdu China; 9 Huadong Hospital, Fudan University Shanghai China; 10 The Affiliated Hospital of Guizhou Medical University Guiyang, Guizhou China

**Keywords:** artificial Intelligence, assist, decision-making, general anesthesia, surgery

## Abstract

**Background:**

AI is rapidly transforming medical practice, with emerging applications in perioperative care and anesthesiology. However, the clinical implementation of AI-assisted decision-making systems in anesthetic management remains challenging and requires comprehensive evaluation.

**Objective:**

This study aimed to assess the performance and clinical applicability of an AI-assisted decision support system (ZW-AA-001) for general anesthesia management by comparing its decisions with those of experienced anesthesiologists across 6 medical centers.

**Methods:**

A multicenter retrospective study was conducted using perioperative data from 1008 patients who underwent elective noncardiac surgeries under total intravenous anesthesia. The AI system’s recommendations for anesthetic and hemodynamic medication adjustments were compared with anesthesiologists’ decisions. Key outcomes included decision concordance rates, temporal performance, and consistency across centers. Advanced statistical methods, including prevalence-adjusted and bias-adjusted κ (PABAK) and Gwet’s first-order agreement coefficient (AC1), were used to evaluate agreement metrics.

**Results:**

The study included 1008 patients, with a median age of 50 (IQR 37-59) years and female predominance (619/1008, 61.4%). During anesthesia maintenance, the AI system demonstrated moderate overall decision agreement with anesthesiologists (73.3%, 95% CI 72.4%-74.3%). Analysis of center-specific data revealed generally consistent performance across all 6 centers. For propofol management, high concordance was observed in dosage adjustment decisions (91.1%, 95% CI 90.4%-91.8%), though fair agreement was found in adjustment direction (68.6%, 95% CI 67.3%-69.8%). The AI system showed significantly faster decision-making time compared to anesthesiologists for the adjustment of propofol (pseudomedian difference –77.5, 95% CI –79.5 to –75.5 seconds; *P*<.001). Although esmolol-related decisions showed a numerically higher concordance of 71.4%, this was not statistically significant (PABAK=0.429; *P*=.21; AC1=0.622; *P*=.09). Decisions for atropine, ephedrine, and urapidil demonstrated substantially lower agreement (17.4%-29.8%). The AI system recommended hemodynamic interventions more frequently than anesthesiologists across all medications.

**Conclusions:**

The AI-assisted decision support system demonstrated varying levels of concordance with anesthesiologists in managing surgery, with the highest agreement observed in propofol administration. However, the system’s lower agreement in hemodynamic medication management highlights the need for further optimization and validation. These findings underscore the potential of AI systems to enhance anesthetic decision-making, improve efficiency, and address workforce challenges in anesthesiology. Future research should focus on expanding the system’s capabilities, validating its performance in prospective clinical trials, and ensuring its integration into diverse clinical settings.

## Introduction

AI has demonstrated remarkable potential to transform contemporary medical practice [1], exhibiting significant capabilities across diverse clinical domains, from diagnostic applications in radiology [2] and pathology [3] to therapeutic and interventional applications in cardiology [4] and surgery [5].

Anesthesiology emerges as an ideal candidate for AI integration, encompassing multiple facets of clinical care, including perioperative management, critical care medicine, pain therapy, pharmacological applications [6], and the education of medical students [7]. The complex nature of anesthesia depth management, influenced by drug pharmacokinetics and individual patient variability, in conjunction with dynamic surgical stimuli, necessitates continuous modulation of sedation and analgesia levels. The implementation of AI-driven high-frequency monitoring and control systems presents opportunities to enhance the precision and stability of anesthetic management through optimized drug titration [8].

The potential impact of AI extends to alleviating critical workforce challenges in anesthesiology. Data from the Chinese Anesthesiology Department Tracking Database (2018-2019) revealed that Chinese anesthesiologists conducted over 4.6 million anesthetic procedures annually between 2015 and 2017. This period witnessed an unprecedented 929% increase in operating room anesthesia cases, alongside a 597% growth in the anesthesiology workforce. Despite this expansion, the average individual workload rose by approximately 10%. This increased workload has been associated with high rates of burnout among clinical anesthesiologists, with prevalence exceeding 68% in certain regions, significantly surpassing rates reported in Western nations [9]. The requirement for sustained vigilance during continuous monitoring of anesthetic depth and drug titration throughout surgical procedures becomes particularly challenging under such high-volume clinical conditions. AI-assisted systems present a promising approach to reduce cognitive burden and minimize variability in care resulting from clinician fatigue and workload demands.

Recent years have witnessed significant advancement in AI applications within perioperative care. These developments encompass preoperative airway assessment for difficult airway prediction [10], computer vision-assisted tracheal intubation guidance [11], and sophisticated risk stratification models [12]. While these AI-driven tools have shown potential to support clinical decision-making and enhance patient safety, research on personalized anesthetic drug administration using open-loop AI algorithms during the maintenance phase of general anesthesia remains limited. Although previous studies have demonstrated the feasibility of automated drug control through open-loop AI models, the concordance between AI-recommended dosing and anesthesiologist-directed administration has varied considerably, ranging from 70% to 94% [13]. Furthermore, these investigations have primarily focused on propofol and remifentanil, with limited evaluation of hemodynamic parameter management, particularly in the regulation of heart rate (HR) and blood pressure.

The general anesthesia AI-assisted decision-making system (ZW-AA-001), developed by Shanghai Ruiyiwei Medical Technology Co, Ltd, China, is designed to optimize anesthesia depth and circulatory management throughout the perianesthetic period. This study aimed to assess the concordance between AI-assisted recommendations and decisions made by experienced anesthesiologists.

## Methods

### Ethical Considerations

The study protocol was approved by the institutional ethics committees of all participating centers (approval numbers 2023-762; k2024-259-00; 2023-173-(1); HXSY-EC-2024005; WCHSIRB-D-2024-152; and 2023153K, respectively). Given the retrospective design and use of deidentified data, the requirement for informed consent was waived. Registration unique identifying number: MR-31-23-023706. This study adhered to the ethical guidelines outlined in the Declaration of Helsinki. Reporting of this trial followed the STROBE (Strengthening the Reporting of Observational Studies in Epidemiology) guidelines [14] and the GRRAS (Guidelines for Reporting Reliability and Agreement Studies) [15].

### Study Design and Population

This multicenter retrospective study analyzed perianesthetic data from 6 medical centers in China, collected from March to December 2024: (1) Beijing Chaoyang Hospital of Capital Medical University; (2) Beijing Jishuitan Hospital of Capital Medical University; (3) Shanghai Sixth People Hospital affiliated to Shanghai Jiao Tong University School of Medicine; (4) West China Fourth Hospital of Sichuan University; (5) West China Hospital of Stomatology, Sichuan University; and (6) The Affiliated Hospital of Guizhou Medical University. The study enrolled patients aged 18 years or older who underwent noncardiac surgery under total intravenous anesthesia, classified as American Society of Anesthesiologists I to III, at the participating centers. All centers used standardized monitoring systems (Mindray BeneVision N Series), intravenous infusion pumps (Medcaptain), and bispectral index (BIS) monitoring (Mindray Monitor BIS module) for anesthesia depth assessment. Exclusion criteria included cases with incomplete or irretrievable real-time monitoring data, infusion pump records, or anesthesiologist documentation. Patients receiving concurrent inhalational anesthetics were also excluded. The centers included in this study were specifically selected for their use of similar anesthesia protocols, and the criteria for promotion to attending anesthesiologist were consistent across all sites.

### Data Extraction

This retrospective analysis used clinical data from existing medical records. Vital signs were recorded at 1-minute intervals using standardized monitoring systems. However, medication administration and adjustment events were recorded with second-level precision to capture the exact timing of these interventions. Monitored parameters included systolic blood pressure (SBP), diastolic blood pressure (DBP), mean arterial pressure (MAP), HR, BIS, and end-tidal carbon dioxide. Intravenous infusion data included baseline infusion rates, rate adjustments, and cumulative medication administration. Analyses focused on the maintenance phase of general anesthesia, defined as the period between initiation and termination of continuous intravenous drug administration. The simulation evaluated the AI system continuously and independently, polling the patient state and logging the AI’s recommendations regardless of human actions. Concordant matches between AI decisions and anesthesiologists’ interventions were defined based on a 300-second (5-minute) temporal window [16], centered on the time point of either an anesthesiologist’s intervention or the AI system’s determination of the need for intervention. While the timing and type of interventions were documented, certain granular details about the decision-making process (such as the exact duration of parameter deviations before intervention) were not consistently available in the historical records. Therefore, our analysis focuses on documented interventions and their outcomes rather than real-time decision-making processes of anesthesia. To maintain data integrity and prevent retrospective bias, we deliberately chose not to have anesthesia experts review the AI decisions. This approach preserved the authenticity of the real-world clinical decisions and ensured that the comparison between AI recommendations and actual clinical practice remained unbiased by post hoc expert opinions.

### General Anesthesia AI-Assisted Decision-Making (Robot) System (ZW-AA-001)

**Figure 1:**
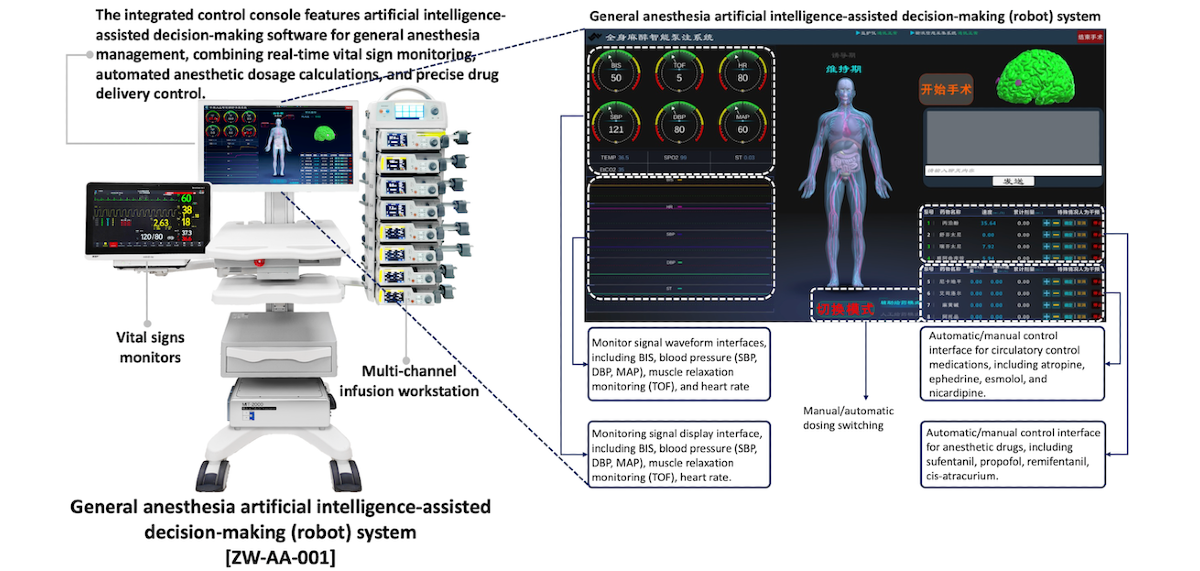
Diagram of the AI-assisted decision-making system under general anesthesia. BIS: bispectral index; DBP: diastolic blood pressure; MAP: mean arterial pressure; SBP: systolic blood pressure; TOF: train-of-four.

The general anesthesia AI-assisted decision-making system (ZW-AA-001; patent number CN 116779152 B [17]; Figure 1) was developed through a collaboration among Shanghai Ruiyi Medical Technology Co, Ltd, the Institute of Automation of the Chinese Academy of Sciences, and the Shanghai Brain Intelligence Engineering Center [18]. This high-performance computing platform processes complex biological signal data to address critical challenges of anesthesiologist workforce shortages and operational standardization (Figure 1). The system uses advanced AI technology, incorporating dynamic real-time monitoring and multimodal signal fusion algorithms to optimize the delivery of general anesthesia.

The system comprises three primary technical modules:

1. Real-time monitoring module: uses high-precision sensors (flexible electrodes and spectral analysis devices); monitors vital parameters including blood pressure, HR, and oxygen saturation; implements AI prediction models (such as long short-term memory networks for time-series forecasting) for early warning of clinical abnormalities.
2. Intelligent control module: the intelligent control module integrates both rule-based and learned decision-making approaches. Fuzzy control algorithms (using predefined membership functions for MAP and BIS deviations) are used to manage predefined safety thresholds, ensuring robust responses under critical conditions. Simultaneously, artificial neural networks (specifically, multilayer perceptrons) dynamically adjust drug infusion rates by learning from patient-specific inputs, allowing for personalized and adaptive control. The system adapts to individual patient characteristics, such as weight, age, and baseline vital signs, by using these parameters as inputs to the artificial neural networks, which optimize drug infusion rates in real time.
3. Federated learning and privacy protection module: implements federated learning (using the federated averaging algorithm) technology for multicenter data training; optimizes algorithm accuracy through collaborative institutional data sharing; and maintains patient privacy protection during data processing. To ensure robustness and generalizability, model training and validation were performed on separate datasets.

Grounded in the principles of “ideal anesthetic state” [19], this AI-assisted decision-making system integrated expert anesthesiologists’ clinical knowledge, professional judgment, and empirical experience into an automated application for precise control of anesthetic agents and vasoactive medications during general anesthesia.

### Anesthesia Management

A similar standardized anesthesia induction protocol was implemented across all centers, including sufentanil, propofol, and cisatracurium. Anesthesia was maintained using continuous propofol infusion. The AI system’s therapeutic targets were guided by established principles of the “ideal anesthesia” [19], aiming to maintain BIS values between 40 and 60 and hemodynamic stability (blood pressure 90-110/60-80 mm Hg, HR 55-80 beats/minute). The administration criteria for vasoactive agents were defined as follows: ephedrine was indicated when either SBP was less than 80 mm Hg with DBP not exceeding 90 mm Hg, or when SBP was at least 80 mm Hg with DBP less than 60 mm Hg and MAP less than 60 mm Hg. Urapidil was administered when SBP exceeded 130 mm Hg with DBP at least 60 mm Hg, or when SBP was between 80 and 130 mm Hg with DBP above 90 mm Hg, or when SBP was at least 80 mm Hg with DBP less than 60 mm Hg and MAP above 110 mm Hg. For HR management, esmolol was administered when HR was at least 90 beats/minute, while atropine was given when HR was at or below 45 beats/minute. All analyzed vasoactive medications (esmolol, atropine, ephedrine, and urapidil) were administered as single-dose boluses during the maintenance phase of general anesthesia. Continuous infusion was not used for these agents in any of the participating centers. Propofol was the only medication administered via continuous intravenous infusion, with infusion rates adjusted dynamically based on the patient’s anesthetic depth and hemodynamic status. Although M-hydroxylamine and methoxamine were administered in certain centers according to local practices, they were explicitly excluded from the final agreement analysis because they were not universally used across all participating centers.

### Outcome Measures

The primary outcome measure was the level of concordance between the AI-assisted decision-making system and anesthesiologists’ operations, assessed through percentage agreement with 95% CIs (overall agreement rate = number of concordant decisions/[total anesthesiologist decisions + total AI decisions – number of concordant decisions] × 100%), prevalence-adjusted and bias-adjusted κ (PABAK), and Gwet’s first-order agreement coefficient (AC1). In this study, the overall concordance rate was calculated as a weighted average of agreement between the AI-assisted system and anesthesiologists across all decision-making categories, with weights assigned based on the frequency of each category in clinical practice. Anesthesiologists’ decisions are used as a reference standard for comparison in this study. These advanced agreement metrics were selected based on their superior mathematical properties in calculating chance-corrected agreement [20,21]. Both PABAK and AC1 demonstrate greater stability in interrater reliability assessment compared to Cohen κ, particularly in settings with prevalence imbalance or uneven category distribution—a crucial consideration given the predominance of drug modifications in current studies [21]. Agreement levels were interpreted using Altman’s guidelines, where values ≤0.20 indicate poor reliability, 0.21-0.40 fair reliability, 0.41-0.60 moderate reliability, 0.61-0.80 substantial reliability, and 0.81-1.00 very good reliability. The secondary outcome was temporal performance, defined as the time difference (in seconds) between AI-generated decisions and those made by anesthesiologists. Although the AI system generated recommendations based on vital signs updated at 1-minute intervals (yielding minute-level time stamps, eg, HH:MM:00), the actual medication administration by the anesthesiologists was recorded by the electronic medical record system with single-second precision (eg, HH:MM:SS). Consequently, the decision-making latency—calculated as the difference between the AI recommendation time stamp and the actual human administration time stamp—is reported with single-second precision.

### Statistical Analysis

To simulate real-world clinical scenarios as closely as possible, we did not apply additional preprocessing steps such as filtering or artifact rejection to the continuous monitoring data before inputting it into the AI system. This decision was made to reflect the conditions under which clinicians typically operate, where raw, unfiltered data from monitoring devices is used to guide decision-making during surgery. The AI system was designed to process this raw data directly, without requiring precleaning, to better align with clinical practice.

Continuous variables were tested for normality using the Shapiro-Wilk test. Data following a normal distribution were reported as mean and SD, whereas nonnormally distributed data were presented as median with IQR. For between-group comparisons, the Student *t* test was applied for normally distributed data and the Mann-Whitney *U* test for nonnormally distributed data. For the analysis of time differences, due to the presence of heavily tied data, the Hodges-Lehmann pseudomedian was reported as the point estimate, along with its 95% CI calculated via the Wilcoxon signed-rank test. In accordance with the guidelines for GRRAS [15], 95% CIs were calculated for percentage agreement, PABAK, and AC1 statistics. Subgroup analysis of center-specific data was conducted across all 6 centers. We prioritized estimation over hypothesis testing by reporting agreement metrics (percentage agreement, PABAK, and AC1) with 95% CIs. For PABAK and AC1, *P* values were calculated to test the null hypothesis that the agreement coefficient equals zero (ie, agreement is purely due to chance). All secondary analyses were considered exploratory and not subjected to multiple comparison correction. All statistical analyses were performed using R software (version 4.4.1; R Foundation for Statistical Computing [22]). Statistical significance was set at a 2-sided *P* value <.05. Sample size was determined by available data without prior power calculation. As such, the results should be interpreted as exploratory.

## Results

### Study Population

This multicenter study enrolled 1008 patients who met the predetermined inclusion and exclusion criteria (Figure S1 in Multimedia Appendix 1). Patient distribution varied across participating centers, with the West China Fourth Hospital (n=332, 32.9%) and the Affiliated Hospital of Guizhou Medical University (n=290, 28.8%) contributing the largest patient cohorts. Demographic and clinical characteristics are summarized in Table 1. The study cohort had a median age of 50 (IQR 37-59) years and a median BMI of 23.5 (IQR 21.3-26.0) kg/m^2^, with female patients comprising 61.4% (n=619) of the population. Minimally invasive surgical approaches, including laparoscopic procedures, were used in 92.2% (n=929) of cases, with abdominal surgeries representing 50.6% (n=510) of all procedures. The median operative duration was 74 (IQR 47-110) minutes.

**Table 1:** Patient demographics and baseline characteristics.

Characteristic		Value (N=1008)
**Study centers, n (%)**		
	Affiliated Beijing Chaoyang Hospital of Capital Medical University	139 (13.8)
	Affiliated Beijing Jishuitan Hospital of Capital Medical University	54 (5.4)
	Shanghai Jiao Tong University Affiliated Sixth People’s Hospital	156 (15.5)
	West China Fourth Hospital, Sichuan University	332 (32.9)
	West China Hospital of Stomatology, Sichuan University	37 (3.7)
	The Affiliated Hospital of Guizhou Medical University	290 (28.8)
Age (years), median (IQR)		50 (37-59)
**Gender, n (%)**		
	Female	619 (61.4)
	Male	389 (38.6)
BMI (kg/m^2^), median (IQR)		23.5 (21.3-26.0)
**Type of surgery, n (%)**		
	Minimally invasive	929 (92.2)
	Open	79 (7.8)
**Location of surgery, n (%)**		
	Thoracic	183 (18.2)
	Upper abdomen	87 (8.6)
	Abdomen	510 (50.6)
	Other	228 (22.6)
Duration of surgery (minutes), median (IQR)		74 (47-110)

Consider each drug modification intervention as an event; the total number of intervention decisions recorded from the AI-assisted decision-making system was 7630, and 6694 interventions were made by anesthesiologists during anesthesia maintenance (Table 2). Propofol adjustments constituted the majority of interventions, with anesthesiologists performing 5996 (89.6%) modifications and the AI-assisted system performing 5463 (71.6%). The distribution of propofol dose modifications was comparable between the two (dose increases: 2955, 44.1% vs 2786, 36.5%; dose reductions: 3041, 45.4% vs 2677, 35.1%, respectively). Interventions with atropine, ephedrine, and esmolol were less frequent throughout the maintenance phase.

**Table 2:** Summary of anesthesiologist- and AI-algorithm–driven drug adjustments.

				Anesthesiologist		AI-assisted decision-making
The total number of adjustments				6694		7630
**The type and dose of the drug**						
	**Propofol**					
		n (%)	5996 (89.6)		5463 (71.6)	
		ml/h^a^, median (IQR)	5.00 (3.00-10.00)		3.60 (2.40-4.74)	
	**Atropine,**					
		n (%)	99 (1.5)		172 (2.3)	
		mg, median (IQR)	0.50 (0.30-0.50)		0.20 (0.20-0.20)	
	**Esmolol**					
		n (%)	7 (0.1)		5 (0.1)	
		mg, median (IQR)	20.00 (15.0-20.0)		10.00 (10.0-10.0)	
	**Ephedrine**					
		n (%)	132 (2.0)		481 (6.3)	
		mg, median (IQR)	6.00 (6.00-6.00)		3.00 (3.00-3.00)	
	**Urapidil**					
		n (%)	460 (6.9)		1509 (19.8)	
		mg, median (IQR)	10.00 (10.00-10.00)		10.00 (7.00-10.00)	
**The type of adjustments^b^, n (%)**						
	Single-dose medication^c^		698 (10.4)		2167 (28.4)	
	Decreased dose^d^		3041 (45.4)		2677 (35.1)	
	Increased dose^d^		2955 (44.1)		2786 (36.5)	

^a^The values reflect the absolute difference between the infusion rate before and after an adjustment.

^b^All analyzed vasoactive medications (esmolol, atropine, ephedrine, and urapidil) were administered as single-dose boluses. Propofol was the only medication administered via continuous intravenous infusion, with infusion rates dynamically adjusted by the AI system.

^c^Refers to vasoactive agents administered as a single bolus.

^d^Refers to propofol administered by continuous pump infusion.

### Outcome: Decision-Making Consistency

**Figure 2:**
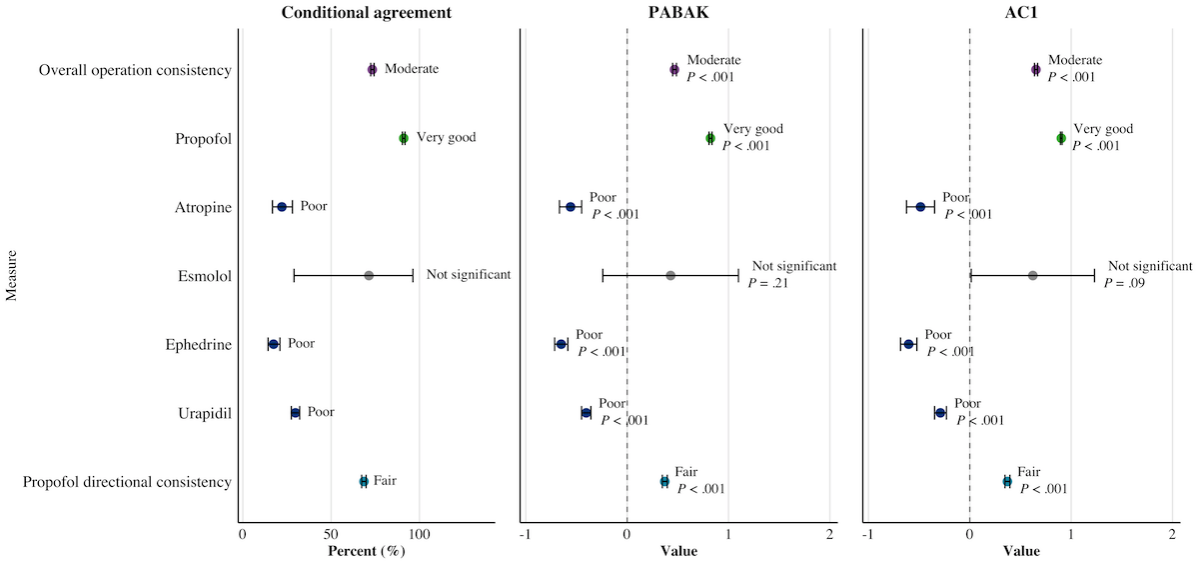
General anesthesia AI-assisted decision-making system and anesthesiologist and consistency results. The left panel shows the raw conditional agreement (percent), while the middle and right panels display the prevalence-adjusted and bias-adjusted κ (PABAK) and Gwet’s first-order agreement coefficient (AC1) along with their respective *P* values. Error bars represent 95% CIs. The reliability descriptors are defined as follows: ≤0.20 (poor), 0.21-0.40 (fair), 0.41-0.60 (moderate), 0.61-0.80 (substantial), and 0.81-1.00 (very good). The agreement for esmolol was not statistically significant and is labeled accordingly; this specific result should be interpreted with extreme caution given the extremely small sample size.

The overall decision agreement between anesthesiologists and the AI-assisted system during anesthesia maintenance achieved 73.3% concordance (the total number of concordant decisions=6060, 95% CI 72.4%-74.3%; Figure 2), indicating moderate agreement. This finding was corroborated by moderate to good decision concordance as measured by PABAK (0.467, 95% CI 0.448-0.486; *P*<.001) and AC1 (0.653, 95% CI 0.638-0.669; *P*<.001). To further address the potential bias introduced by the large sample size and high consistency of propofol, we performed a weighted pooled analysis excluding propofol. For the remaining vasoactive medications, the adjusted overall consistency dropped to 26.3% (95% CI 24.5 to 28.2). Similarly, the pooled PABAK and AC1 values for these drugs were –0.474 (95% CI –0.510 to –0.437; *P*<.001) and –0.378 (95% CI –0.424 to –0.332; *P*<.001), respectively.

**Figure 3:**
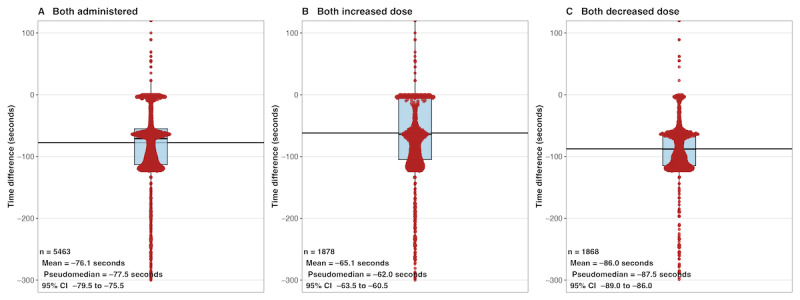
Time difference between the AI-assisted decision-making system of general anesthesiologists and the adjustment of propofol. While the AI system indeed relies on vital signs updated at 1-minute intervals (meaning the AI generates a recommendation exactly at the minute mark, eg, HH:MM:00), the actual medication administration by the anesthesiologist is logged by the electronic anesthesia information system with single-second precision (eg, HH:MM:SS). Due to the skewed and heavily tied nature of the temporal data, the Hodges-Lehmann pseudomedian is reported as the point estimate to ensure it is correctly bounded by the 95% CI. The latency is calculated as the exact difference between these 2 time stamps: latency = (AI recommendation time stamp [minute-level]) – (actual doctor administration time stamp [second-level]).

Analysis of propofol administration revealed differential levels of agreement across decision domains. The binary decision regarding propofol dosage adjustment demonstrated excellent concordance (the total number of concordant decisions=5463, 91.1%, 95% CI 90.4%-91.8%), supported by robust PABAK (0.822, 95% CI 0.808-0.837; *P*<.001) and AC1 (0.903, 95% CI 0.894-0.911; *P*<.001) values. In contrast, concordance on the direction of dose adjustment was fair (the total number of concordant decisions=3746, 68.6%, 95% CI 67.3%-69.8%), and corresponding PABAK and AC1 values of 0.371 (95% CI 0.347-0.396; *P*<.001) and 0.371 (95% CI 0.347-0.396; *P*<.001), respectively. Temporal analysis of the propofol decision-making time revealed significantly shorter decision-making latency for the AI-assisted system compared to anesthesiologists (pseudomedian difference: –77.5, 95% CI –79.5 to –75.5 seconds; *P*<.001; Figure 3). This pattern persisted for both propofol rate increases and decreases, with pseudomedian differences of –62.0 (95% CI –63.5 to –60.5; *P*<.001) and –87.5 (95% CI –89.0 to –86.0; *P*<.001) seconds, respectively. Analysis of center-specific data revealed generally consistent performance across all 6 centers. Although some variations in the magnitude of concordance were observed, along with reduced statistical power in smaller subgroups, the overall performance remained largely consistent across different subgroups. The overall concordance rates ranged from 49.8% to 94.2% (Table S1 in Multimedia Appendix 1). Propofol dosage adjustment showed particularly stable performance across centers (concordance range 86.5%-95.6%), while propofol directional consistency exhibited more variation (concordance range 59.5%-73.5%). Our key conclusions are based on effect estimates and their CIs for agreement metrics and remain unchanged after applying multiplicity control.

**Figure 4:**
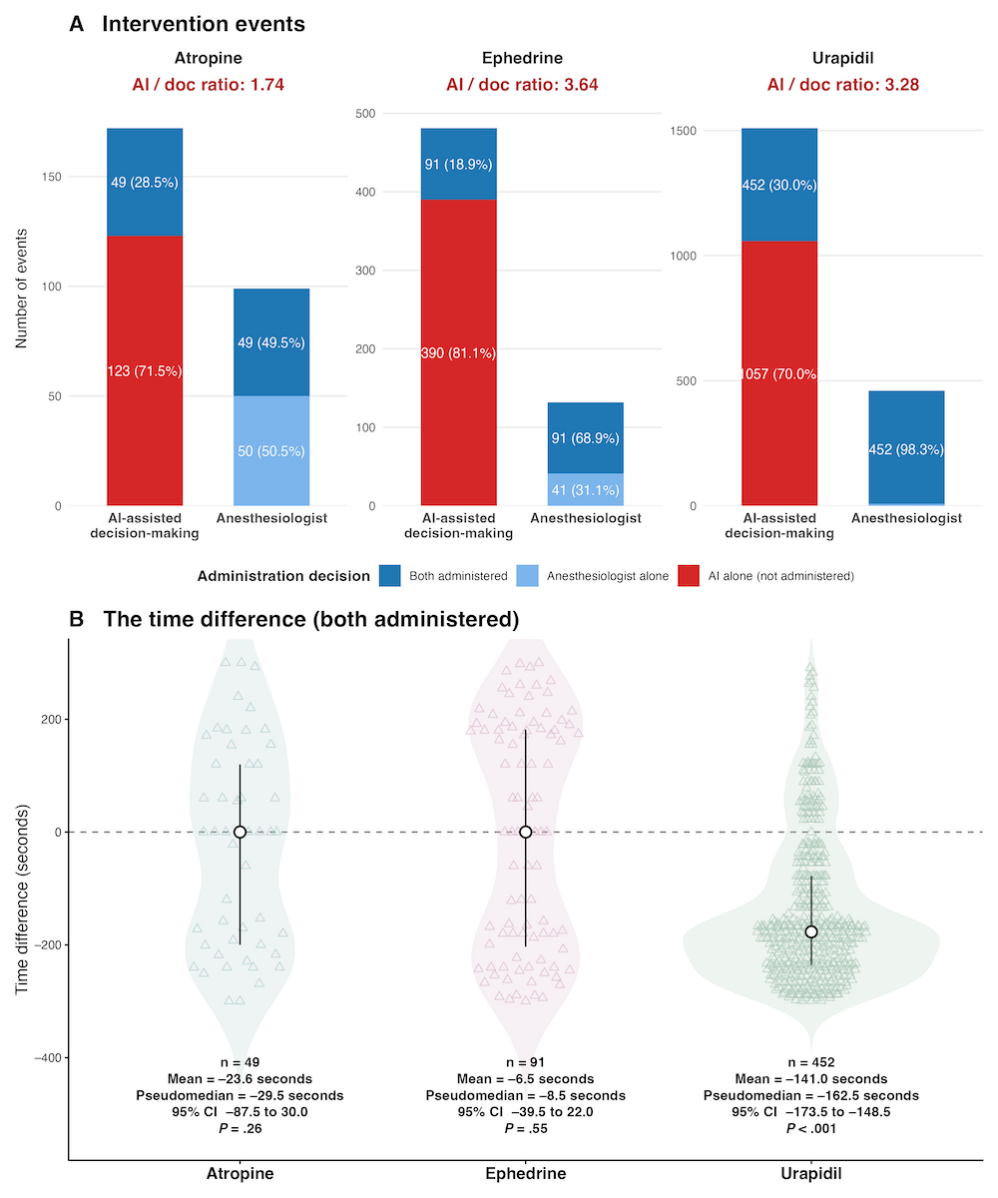
The (A) percentage and (B) time difference between the AI-assisted decision-making system of general anesthesiologists and the adjustment of vasoactive drugs. The AI/doctor (Doc) Ratio is calculated as the total number of AI administration decisions divided by the total number of anesthesiologist administration decisions. The stacked bars represent the absolute event counts corresponding to Table 2 (eg, for Urapidil: AI total=1057 administered alone + 452 both=1509; anesthesiologist total=8 administered alone + 452 both=460). The percentages within the bars are calculated using their respective column totals (ie, the total number of AI recommendations or the total number of anesthesiologist interventions) as the denominator. The total number of unique events where at least one party (AI or anesthesiologist) administered the drug is 222 for Atropine, 522 for Ephedrine, and 1517 for Urapidil. In panel B, the Hodges-Lehmann pseudomedian is reported as the point estimate for time differences.

Concordance in hemodynamic medication management varied substantially across different agents. None of the vasoactive drugs demonstrated statistically significant agreement; while esmolol reached a concordance of 71.4% (PABAK=0.429; *P*=.21; AC1=0.622; *P*=.09), decisions regarding atropine, ephedrine, and urapidil showed substantially lower concordance (17.4%-29.8%; Figure 2). The interpretation of our findings regarding esmolol interventions requires caution due to the limited number of adjustments observed in our study. This small sample size may not provide sufficient statistical power to draw robust conclusions about the AI system’s performance in hemodynamic medication management. The AI-assisted system recommended approximately 1.74 to 3.64 times more interventions for atropine, ephedrine, and urapidil than anesthesiologists (Figure 4A). In no scenario did the proportion of anesthesiologist interventions exceed that of the AI system. While response times were comparable for atropine (*P*=.26) and ephedrine (*P*=.55), anesthesiologists exhibited significantly longer latency in urapidil administration (pseudomedian difference –162.5, 95% CI –173.5 to –148.5 seconds; Figure 4B).

## Discussion

### Principal Findings

This real-world data-based study evaluated an AI-assisted decision-making system for anesthesia management and revealed varying levels of concordance with anesthesiologists. Notably, the system demonstrated the highest agreement in the regulation of anesthesia depth during propofol administration, despite some differences in consistency across other areas. The system also showed reduced intervention latency compared with clinician-directed decisions. While agreement was lower for cardiovascular drug administration, this likely reflected the AI system’s more proactive approach to hemodynamic management rather than a deficiency in decision-making capability. While the AI system demonstrates high agreement with anesthesiologists, this does not necessarily validate its clinical appropriateness or safety.

### Comparison to Prior Work

Previous research has primarily focused on isolated or limited combinations of anesthetic monitoring parameters [23]. A notable multicenter study evaluating BIS-guided closed-loop anesthesia delivery systems demonstrated superior performance in maintaining target BIS ranges compared with manual control (81.4%, SD 8.9% vs 55.3%, SD 25.0% of total anesthesia time) [24]. However, comprehensive assessments of AI applications spanning the entire perioperative period remain limited. Ren et al [13] investigated the feasibility of implementing an intelligent control system for anesthetic drug delivery during the maintenance phase of general anesthesia. Their system, based on a convolutional neural network with an open-loop design, was trained on established anesthesia protocols using machine learning algorithms. The automated drug dosing decisions generated by the AI system were found to be comparable to those made by experienced clinical anesthesiologists during the maintenance phase. However, when evaluated using a cumulative error threshold of 0.02 µg/kg/min, the model achieved a sampling precision of only approximately 70%, indicating the need for further refinement. Despite these limitations, this study marked a significant milestone in validating AI-assisted anesthesia management and laid the groundwork for future development. Building on this foundation, our study introduces novel advances in AI-based anesthetic management, particularly through the integration of cardiovascular monitoring and control, representing a significant step toward fully automated anesthesia management.

The implementation of AI in clinical settings presents substantial challenges [25]. Hager et al [26], using the Medical Information Mart for Intensive Care database with 2400 real patient cases across 4 common abdominal pathologies, revealed significant limitations in current AI models. The results demonstrated that these models consistently underperformed relative to physician-level diagnostic accuracy and failed to adhere to established diagnostic and treatment guidelines. Moreover, the inability of these systems to accurately interpret laboratory results raises serious concerns regarding patient safety. Implementation challenges extend beyond diagnostic capabilities, as these models often exhibit inconsistent adherence to instructions and are highly sensitive to both the quantity and sequence of input information, complicating their integration into existing clinical workflows. These limitations are especially critical in anesthesiology, where decision-making requires real-time integration of complex physiological parameters. To address these challenges, we developed our AI-assisted system using a robust multicenter database encompassing over 1000 patients and 6000 anesthetic interventions, ensuring alignment with established clinical practices while minimizing algorithmic bias. These records capture diverse clinical scenarios and decision-making patterns from experienced anesthesiologists, serving as a robust foundation for both AI-assisted system development and validation. This robust data-driven approach emphasizes adherence to established clinical practice and aims to minimize algorithmic bias, ensuring that system-generated recommendations align closely with expert-level care.

Our trial demonstrated favorable outcomes, particularly in propofol management, where the AI-assisted decision-making system achieved remarkable concordance (>90%) with anesthesiologists’ decisions. These results validate previous research on the use of BIS and blood pressure parameters for anesthesia depth regulation [24]. In contrast, lower concordance was observed in hemodynamic management, which appears to reflect methodological differences rather than intrinsic limitations of the AI system. This system adopted a more proactive intervention strategy, initiating hemodynamic interventions more frequently than anesthesiologists and maintaining strict adherence to predetermined protocols. Notably, clinical guidelines recommend maintaining perioperative MAP above 65 mm Hg [27]. However, observational data showed that anesthesiologists often initiate vasopressor therapy at lower thresholds, with mean intervention levels typically at SBP below 90 mm Hg or MAP below 65 mm Hg [28]. This suggests that anesthesiologists tend to use a more flexible approach, tolerating greater hemodynamic variability rather than strictly adhering to guideline-recommended thresholds. Such practice, while pragmatic, merits careful consideration. Previous studies have provided high-quality evidence demonstrating that intraoperative hypotension (MAP <60-70 mm Hg or SBP <90-100 mm Hg) is significantly associated with serious adverse outcomes, including acute kidney injury, myocardial injury, myocardial infarction, and mortality [27,29]. The risk of these complications is directly correlated with both the severity and duration of hypotensive episodes. In contrast, the AI-assisted decision-making system implements more systematic interventions based on strict adherence to predefined thresholds, potentially offering more consistent and guideline-compliant hemodynamic control. The discrepancies observed between AI decisions and anesthesiologists’ interventions may be attributed to several factors [30]: limitations in the retrospective documentation of anesthesia records, which may contain timing inaccuracies or post hoc modifications, or current limitations of our AI algorithm in capturing the nuanced clinical judgment that experienced anesthesiologists use. While this algorithmic approach may enhance the safety and rigor of intraoperative management, the observed higher intervention frequency of the AI system requires careful consideration of both benefits and potential risks, such as drug overuse or patient discomfort. However, the AI system primarily relies on specific parameters such as SBP and BIS values, which may oversimplify the complexity of anesthesia management. Effective anesthesia requires integrating multiple factors, including patient condition, laboratory results, and clinical context, which the AI system cannot fully replicate. Anesthesiologists’ expertise and judgment remain essential for ensuring patient safety, especially in complex and dynamic scenarios. Therefore, its clinical significance and potential benefits require confirmation through prospective clinical trials.

Although our analysis showed differences between AI and manual interventions, the underlying decision-making processes remain unclear from the available data. These differences likely stem from varying intervention thresholds and approaches to maintaining stability. A key point of our AI system is its reliance on standardized vital sign ranges [19], which contrasts with the individualized approach of experienced anesthesiologists who adjust targets based on patient-specific factors. Moreover, AI disagreement with anesthesiologists does not always indicate an error. In some cases, it may represent proactive management strategies based on real-time data that differ from human decision-making. It is worth noting that while our results show promising consistency rates, the AI system’s performance was evaluated in a simulated environment. We recognized that AI-assisted drug administration decisions need to clearly define the roles and responsibilities between the AI system and anesthesiologists, particularly in high-risk scenarios [31]. The current AI system functions strictly as a decision support tool, not as an autonomous decision-maker. In critical situations such as severe hypotension (MAP <60 mm Hg) or extreme BIS values (<30 or >80), the system maintains its advisory role while the anesthesiologist retains full authority and responsibility for clinical decisions. The AI system’s processing logic is fundamentally based on preprogrammed algorithms and does not supersede human clinical judgment. It lacks the capability to integrate complex contextual factors such as patient-specific risk factors and comorbidities, or surgical field conditions and communication with surgeons. This limitation underscores that the AI system should serve as a supplementary tool to enhance, rather than replace, the anesthesiologist’s clinical expertise [6,31]. The final responsibility for patient safety and clinical decisions must remain with the human physician. Future implementation of AI-assisted systems in anesthesia requires clear institutional protocols defining the scope and limitations of AI assistance, explicit documentation of decision-making authority, regular evaluation of system performance and safety metrics [32]. These ethical and legal considerations should be addressed through professional guidelines and regulatory frameworks before widespread clinical implementation [32,33].

### Strengths and Limitations

This study demonstrated several notable strengths, particularly its real-world setting and comprehensive evaluation of decision-making patterns between AI-assisted system and anesthesiologists in the management of commonly used anesthetic agents and hemodynamic medications. However, several important limitations should be acknowledged. First, as a retrospective simulation-based analysis, our study cannot directly assess the actual clinical impact of the AI’s recommendations. Important surrogate metrics (eg, time within target MAP/BIS ranges) and clinical end points (eg, actual drug consumption, hemodynamic stability, and adverse events) could not be evaluated. These dynamic physiological responses must be thoroughly investigated in future prospective trials. Furthermore, the lack of data preprocessing, such as artifact rejection, may have introduced noise and potentially impacted the AI system’s performance [30,34]. This limitation was an intentional design choice to emulate real-world clinical practice, where clinicians rely on raw monitoring data without additional filtering. Besides, the retrospective nature of our study limits the identification of true negatives, where both the anesthesiologist and the AI system correctly decided not to intervene. This exclusion is acknowledged as a limitation and may affect the calculation of agreement metrics such as AC1 [35]. Specifically, because the vast majority of intraoperative time consists of “true negatives” (ie, no intervention needed), excluding these periods likely underestimates the absolute overall agreement rate. By evaluating agreement only during active intervention windows or moments of disagreement, our metrics (PABAK and AC1) are calculated without a complete decision space. This makes our reported reliability scores more conservative, though arguably more reflective of clinically challenging moments rather than baseline stability. Despite this, our analysis provides insights into the clinical decision-making process, particularly in cases of urapidil and ephedrine, where AI-recommended interventions were not acted upon by clinicians, highlighting potential areas of clinical tolerance and variability. Future research should aim to address this limitation by capturing true negatives in prospective studies. Second, the study design introduces a potential ground truth fallacy by treating retrospective human decisions as the infallible standard for concordance. As anesthesiologists often deviate from clinical guidelines by tolerating lower blood pressure thresholds, the AI system, which strictly adheres to predefined physiological thresholds, may have been penalized in the concordance score for being more guideline-compliant [30]. This limitation underscores the need for independent clinical adjudication to evaluate the safety and appropriateness of decisions made by AI and clinicians in cases of disagreement. Additionally, the lack of predictive clinical judgment and situational awareness of the surgical field by the AI system may partly explain the observed discrepancies in intervention timing and frequency. Future system improvements should focus on integrating predictive capabilities and contextual awareness to enhance alignment with human clinical judgment. Third, the study’s reliance on retrospective human decisions as the gold standard for concordance introduces a potential bias. Without independent clinical adjudication, it is challenging to determine objectively which decisions, those made by the AI system or human clinicians, were safer or more appropriate. This limitation is particularly relevant given that anesthesiologists may deviate from clinical guidelines based on their predictive judgment and situational awareness, factors that the AI system currently lacks. Fourth, the overall concordance rate (73.3%, 95% CI 72.4%-74.3%) was substantially influenced by the high agreement in propofol management (91.1%, 95% CI 90.4%-91.8%). This influence should be considered when interpreting the system’s overall performance, as the composite agreement rate may not fully reflect the varying levels of concordance across different decision categories. When excluding propofol from the analysis, the alternative overall concordance for the remaining medications was poor. This subanalysis confirms that while the AI algorithm performs exceptionally well in sedative adjustments, its concordance with anesthesiologists regarding vasoactive drug interventions remains suboptimal. Additionally, the large SD in propofol adjustments reported for the AI system reflects a major clinical conflict where, in many instances, one party recommended increasing anesthesia depth while the other recommended decreasing it. Such conflicts could severely impact patient safety in a real-world setting. This discrepancy is partly due to the weight-based fixed-rate infusion mode used in this study, which contributes to the observed variability as the magnitude of adjustments is influenced by patient body weight. While the system incorporates basic pharmacokinetic principles, it does not fully replicate the complexity of standard target-controlled infusion models. This limitation may contribute to the observed discrepancies and highlights the need for further refinement of the AI system to enhance its clinical use and alignment with established practices. In addition, the relatively infrequent use of vasoactive agents in routine anesthesia practice limits the power of the current analysis, highlighting the need for larger cohorts to more robustly assess the AI system’s accuracy in this domain. The AI system recommended approximately 1.74 to 3.64 times more hemodynamic interventions than anesthesiologists. While this finding suggests that the AI may be more proactive in maintaining hemodynamic stability, its clinical implications remain unclear. Specifically, the potential benefits of reducing hypotension burden must be weighed against possible risks, such as increased drug exposure or oscillatory control. Furthermore, while the AI system’s recommendations align with current clinical guidelines, it is essential to evaluate whether this level of intervention is clinically appropriate or excessive [30]. Future research should include a sensitivity analysis examining the effects of threshold strictness and intervention frequency on patient outcomes, as well as the broader implications of AI-driven decision-making in anesthesia management. Fifth, despite incorporating train-of-four monitoring capabilities, our system’s evaluation did not include neuromuscular blocking agents. Given the diverse range of muscle relaxants used in clinical practice (including cisatracurium, vecuronium, and rocuronium), future studies are needed to address the unique challenge posed by neuromuscular blockade management. Moreover, the use of predefined hemodynamic thresholds in the AI system, while grounded in the concept of “ideal anesthesia,” presents challenges in generalizability across diverse patient populations. Anesthesiologists often individualize hemodynamic targets based on patient-specific factors, including comorbidities, surgical context, and physiological trajectories. Strict adherence to fixed thresholds may not always align with optimal clinical decision-making in such scenarios. These limitations underscore the need for future iterations of the AI system to incorporate patient-specific factors and adaptive algorithms that can dynamically adjust thresholds based on real-time physiological and contextual data. In addition, prospective studies are necessary to validate the safety and efficacy of predefined thresholds and to explore the clinical implications of strict guideline adherence versus individualized care. Such efforts will be critical to ensuring the safe and effective deployment of AI-assisted systems in diverse clinical settings. Finally, our study showed improved efficiency with AI assistance, but efficiency gains do not automatically mean better clinical outcomes [36,37], such as hypotension duration, BIS variability, drug consumption, and adverse events, which is a limitation. While this study provides valuable insights into the reliability of the AI system, future prospective studies are necessary to evaluate its impact on physiological stability and patient outcomes.

### Future Directions

The observed discrepancy between the high binary concordance (91.1%, 95% CI 90.4%-91.8%) and the lower directional concordance (68.6%, 95% CI 67.3%-69.8%) in propofol adjustments highlights fundamental differences in decision-making between the AI system and anesthesiologists. Crucially, opposing directional decisions, such as the AI recommending an increase in anesthesia depth while the clinician actively decreases it, carry significant safety implications. If followed blindly, such algorithmic recommendations could lead to unintended oversedation (hemodynamic collapse) or undersedation (intraoperative awareness). While the AI system relies on predefined algorithms to optimize anesthesia depth using real-time physiological data, clinicians often incorporate additional contextual factors, such as the surgical stage and individual patient characteristics. Addressing these divergences and ensuring human-in-the-loop oversight is critical to ensuring patient safety in real-world settings. To minimize potential risks, future efforts should focus on developing robust protocols to manage conflicts between AI recommendations and clinician judgment. These protocols could include real-time alerts, advanced decision-support systems, and enhanced training programs to ensure alignment between AI-driven and human decision-making, always prioritizing patient outcomes [34]. Further research should aim to expand the AI system’s capabilities to encompass the management of neuromuscular blocking agents and analgesics, facilitating a more comprehensive approach to intraoperative pharmacologic decision-making. Additionally, prospective studies are needed to evaluate the impact of AI-driven systems on patient outcomes and to identify specific scenarios where AI assistance can provide the greatest clinical benefit. Importantly, the use of prospective designs and the inclusion of independent expert panels in future studies will be critical to accurately assess the clinical impact and safety of AI-assisted decision-making systems.

### Conclusion

AI-assisted decision-making systems for general anesthesia hold considerable promise in transforming perioperative care through diverse applications in anesthesiology. While our study highlights the feasibility of such systems in anesthetic management, several important challenges remain to be addressed. The evaluation of complex medication regimens, including neuromuscular blocking agents and analgesics, represents a critical area for future investigation. As AI technologies continue to advance, their integration into clinical practice must be guided by careful consideration of safety, efficacy, and the indispensable role of human expertise. Future studies should focus on expanding system capabilities, validating performance across diverse clinical scenarios, establishing standardized protocols while addressing ethical and legal considerations to facilitate responsible real-world implementation, and rigorously evaluating the system’s impact on patient outcomes.

## Appendix

Multimedia Appendix 1 Flowchart of patient enrollment and evaluation of drug adjustment consistence between anesthesiologist and AI algorithm-driven by different centers.

## Abbreviations

AC1: Gwet’s first-order agreement coefficient
BIS: bispectral index
DBP: diastolic blood pressure
GRRAS: Guidelines for Reporting Reliability and Agreement Studies
HR: heart rate
MAP: mean arterial pressure
PABAK: prevalence-adjusted and bias-adjusted κ
SBP: systolic blood pressure
STROBE: Strengthening the Reporting of Observational Studies in Epidemiology

## Data Availability

Deidentified data will be made available upon reasonable request to the corresponding author, subject to approval by the relevant ethics committees. To ensure transparency, a detailed description of the algorithm’s structure and decision-making logic is included in the manuscript. Independent researchers and institutions are encouraged to contact the corresponding author to request access for external validation or audit purposes, in accordance with ethical and legal requirements. Additional information about the AI-assisted decision-making system (ZW-AA-001) can be found on the product website [17].
