# Supplementary material for: Clinical Evaluation of an AI-Assisted Decision Support System for General Anesthesia Management Based on Data From 6 Centers: Comparative Study
Source: J Med Internet Res. 2026 Jul 20;28:e90023. doi: 10.2196/90023 (PMC13434251; doi:10.2196/90023)
Supplement: Supplementary file 1 [file jmir_v28i1e90023_app1.docx]

**Supplementary Materials**

[**Figure S1.** Flow chart of patient enrollment.	2](#_Toc224914599)

[**Table S1.** The evaluation of drug adjustment consistence between anesthesiologist and AI algorithm-driven by different centers.	3](#_Toc224914600)

# **Figure S1.** Flow chart of patient enrollment.

# **Table S1.** The evaluation of drug adjustment consistence between anesthesiologist and AI algorithm-driven by different centers.

| **Indication** | **Percent and 95% confidence interval (CI)** | **PABAK** | | **AC1** | |
| --- | --- | --- | --- | --- | --- |
|  |  | **Value** | **P** | **Value** | **P** |
| ***Affiliated Beijing Chaoyang Hospital of Capital Medical University*** | | | | | |
| Overall consistency (yes or no) | 53.6% [49.8% - 57.3%] | 0.072 [-0.002 - 0.145] | .06 | 0.279 [0.200 - 0.358] | <.001 |
| Propofol adjustment | 86.5% [82.9% - 89.6%] | 0.730 [0.665 - 0.796] | <.001 | 0.846 [0.804 - 0.888] | <.001 |
| Propofol directional consistency | 73.5% [68.7% - 77.9%] | 0.470 [0.380 - 0.560] | <.001 | 0.485 [0.394 - 0.576] | <.001 |
| ***Affiliated Beijing Jishuitan Hospital of Capital Medical University*** | | | | | |
| Overall consistency (yes or no) | 49.8% [43.6% - 56.0%] | -0.004 [-0.125 - 0.117] | .95 | 0.196 [0.059 - 0.332] | .005 |
| Propofol adjustment | 88.1% [81.6% - 92.9%] | 0.762 [0.656 - 0.868] | <.001 | 0.866 [0.799 - 0.933] | <.001 |
| Propofol directional consistency | 59.5% [50.4% - 68.2%] | 0.190 [0.019 - 0.362] | .03 | 0.191 [0.018 - 0.363] | .03 |
| ***Shanghai Jiao Tong University Affiliated Sixth People’s Hospital*** | | | | | |
| Overall consistency (yes or no) | 94.2% [92.9% - 95.4%] | 0.885 [0.861 - 0.909] | <.001 | 0.939 [0.926 - 0.953] | <.001 |
| Propofol adjustment | 95.2% [93.9% - 96.3%] | 0.904 [0.881 - 0.927] | <.001 | 0.950 [0.937 - 0.962] | <.001 |
| Propofol directional consistency | 65.6% [63.0% - 68.2%] | 0.312 [0.261 - 0.364] | <.001 | 0.314 [0.262 - 0.365] | <.001 |
| ***West China Fourth Hospital, Sichuan University*** | | | | | |
| Overall consistency (yes or no) | 75.2% [73.7% - 76.6%] | 0.503 [0.474 - 0.533] | <.001 | 0.683 [0.660 - 0.706] | <.001 |
| Propofol adjustment | 90.6% [89.3% - 91.8%] | 0.812 [0.788 - 0.836] | <.001 | 0.897 [0.883 - 0.911] | <.001 |
| Propofol directional consistency | 71.6% [69.6% - 73.5%] | 0.432 [0.394 - 0.471] | <.001 | 0.433 [0.395 - 0.472] | <.001 |
| ***West China Hospital of Stomatology, Sichuan University*** | | | | | |
| Overall consistency (yes or no) | 93.1% [90.5% - 95.2%] | 0.862 [0.817 - 0.907] | <.001 | 0.926 [0.900 - 0.952] | <.001 |
| Propofol adjustment | 95.6% [93.2% - 97.3%] | 0.911 [0.872 - 0.950] | <.001 | 0.954 [0.932 - 0.975] | <.001 |
| Propofol directional consistency | 59.5% [54.6% - 64.3%] | 0.190 [0.095 - 0.285] | <.001 | 0.226 [0.127 - 0.325] | <.001 |
| ***The Affiliated Hospital of Guizhou Medical University*** | | | | | |
| Overall consistency (yes or no) | 60.3% [57.9% - 62.7%] | 0.206 [0.159 - 0.253] | <.001 | 0.418 [0.371 - 0.465] | <.001 |
| Propofol adjustment | 87.6% [85.5% - 89.5%] | 0.751 [0.712 - 0.791] | <.001 | 0.859 [0.834 - 0.884] | <.001 |
| Propofol directional consistency | 69.3% [66.2% - 72.2%] | 0.386 [0.327 - 0.444] | <.001 | 0.386 [0.327 - 0.444] | <.001 |

PABAK: prevalence-adjusted and bias-adjusted kappa; AC1: Gwet's first-order agreement coefficient.

The PABAK or AC1 values ≤0.20 indicate poor reliability, 0.21-0.40 fair reliability, 0.41-0.60 moderate reliability, 0.61-0.80 substantial reliability, and 0.81-1.00 very good reliability.
